# Mortality rate, main causes of death and factors associated with higher mortality hazard in late stage Parkinson's disease

**DOI:** 10.1177/1877718X261463369

**Published:** 2026-06-26

**Authors:** Kristina Rosqvist

**Affiliations:** 1 Division of Neurology, Department of Clinical Sciences Lund, 59568 Lund University, Lund, Sweden; 2 Department of Neurology, Skåne University Hospital, Lund, Sweden

**Keywords:** Parkinson's disease, mortality, late stage, cause of death, predictors, mortality hazard rate, dysphagia, aspiration pneumonia

## Abstract

**Background:**

A cohort of 107 patients in late stage Parkinson's disease (PD) was followed for a decade.

**Objective:**

To assess mortality rate, main causes of death and factors associated with higher mortality hazard.

**Methods:**

Late stage PD was defined as Hoehn and Yahr IV-V in “on” or ≤ 50% independency in activities of daily living (ADL). Medical records and mortality records were used to obtain data on mortality.

**Results:**

After three years from baseline, 52 (54%) of the participants were alive, after five years 30 (31%) and after seven years 14 (15%). The most common main cause of death was aspiration pneumonia (34%), followed by sepsis (16%), unspecified pneumonia (13%), ileus (5%) and Covid-19 (5%). Baseline variables significantly associated with higher mortality hazard were worse ADL independency (*P* < 0.001), worse Hoehn and Yahr stage (*P* < 0.001), worse motor function (*P* < 0.001), worse cognition (*P* < 0.001) higher age (*P* = 0.005), worse perceptual problems/hallucinations (*P* = 0.020) and having moved to a nursing home (*P* = 0.021). In a multivariable model, worse motor function (*P* = 0.001), worse depressive symptoms (*P* = 0.023) and worse cognition (*P* = 0.042).

**Conclusions:**

The present end-of-life data provide enhanced knowledge on factors associated with higher mortality hazard in late stage PD. As aspiration pneumonia is the most common cause of death in PD worldwide and swallowing difficulties with the risk of aspiration are common in late stage PD, enhanced focus on prevention and treatment is essential. This may contribute to a better understanding of and care for late stage PD.

## Plain language summary

Death rate, main causes of death and factors associated with higher risk for death in late stage Parkinson’s disease

We followed 107 patients in late stage Parkinson’s disease (PD) for the past decade. The aim of the present study was to assess death rate, main causes of death and factors statistically associated with higher risk for death in late stage PD. Late stage PD was defined as stages IV-V on a disease severity scale (Hoehn & Yahr) ranging I-V, where higher number means worse severity or ≤ 50% independency in activities of daily living (ADL). Medical records and death records were used to obtain data concerning death. Three years from baseline assessment just above half of the participants were still alive, after five years about 30% and after seven years 15%. The most common main cause of death was aspiration (that is, when food, drink or saliva enters the airways) pneumonia (34%), followed by sepsis (16%) and unspecified pneumonia (13%). In statistical analyses, baseline variables significantly associated with higher risk for death were worse ADL independency, worse disease severity (Hoehn & Yahr) stage, worse motor function, worse cognition, higher age, worse perceptual problems/hallucinations and having moved to a nursing home. In a multivariable statistical model, worse motor function, worse depressive symptoms and worse cognition were significantly associated with higher risk for death. The present study provides enhanced knowledge on factors associated with higher risk for death in late stage PD. As aspiration pneumonia is the most common cause of death in PD worldwide and swallowing difficulties with the risk of aspiration are common in late stage PD, enhanced focus on prevention and treatment is essential. Knowledge on death rates, main causes of death and factors associated with higher risk for death may contribute to better understanding of and care for late stage PD.

## Introduction

Late stage Parkinson's disease (PD) constitutes the last years of the patient's life and is a time when the patient has an increased disease burden and there is an increased need of care from both informal family caregivers and the health and social care systems.^1,2^ Research efforts have been initiated over the past decade to increase focus on this severely affected patient group, which is often excluded in research.^2^ It has been shown that there is a high prevalence of motor impairment as well as non-motor symptoms (NMS) burden,^1–5^ which is associated with reduced quality of life (QoL) for both patients and their informal family caregivers.^6–10^ The results have further shown that treatment is often not optimized and could be improved, as therapeutic gains may be reached also in late stage PD.^2,11,12^ To have a movement disorder specialist as treating physician^6^ and to optimize treatment in the late stage may benefit the patient's symptomatology,^11,13,14^ as there is still a good levodopa effect in at least half of the patients.^3^ Palliative care models have been proposed as part of mapping and individualizing patients’ needs in the late stage, as well as for proactive advance care planning together with the patient for the late stage and end-of life related situations.^15–18^ It has been suggested that patients live about 4–5 years in the last disease stage and that hallucinations, regular falls, dementia and need for residential care have been indicated as milestones that occur during this period.^19,20^ Previous studies have shown a varying but increased mortality in PD compared to controls.^21–23^ Advancing age and dementia have been identified across previous studies as the factors most commonly associated with increased mortality.^21^

Knowledge on the most common causes of death and factors associated with higher mortality hazard may aid clinicians to better identify problem areas that can be monitored and addressed in order to improve the symptomatology and the overall situation for the late stage patient.

Through the Care of Late Stage Parkinsonism (CLaSP) project,^2,24^ we have followed a Swedish cohort of 107 late stage PD patients prospectively during the past decade. The aim of the present study was to carry out a long-term assessment and follow-up of this late stage cohort, with focus on investigating mortality rate, main causes of death and factors associated with higher mortality hazard.

## Materials and methods

### Participants and recruitment

Late stage PD was defined as Hoehn and Yahr (HY) stages IV-V (score range I-V, higher = worse)^25^ in the medication “on” state or having a substantial need of help with activities of daily living (ADL), ≤ 50% on the Schwab and England ADL Scale (score range 0–100, higher = better),^26^ which was set as the inclusion criteria. Furthermore, participants should be diagnosed with idiopathic PD since at least 7 years. The diagnosis idiopathic PD was made according to the UK PD Society Brain Bank clinical diagnostic criteria.^27^

Exclusion criteria were cognitive symptoms that started before the PD diagnosis as well as symptomatic parkinsonism such as drug-induced parkinsonism or normal pressure hydrocephalus. The present cohort constituted the Swedish part of the European multicenter CLaSP project.^24^ The present analyses were specific to the Swedish substudy.

### Procedure and clinical evaluation

The participants were recruited from the southern region of Sweden through neurology departments and the municipality-based health care system. An extensive data collection was carried out through home visits. After baseline assessment, the medical records were reviewed for mortality at the time of follow-up visits during the first 18 months after inclusion (at 6, 12 and 18 months) and thereafter mortality was assessed yearly as well as 7–9 years after first inclusion, i.e., all participants were assessed at a final time point 7–9 years after their baseline assessment, as baseline assessments were carried out during approximately a year and a half.

Medical records and mortality records were used to obtain data on mortality. All causes of death were derived from the medical records. Mortality records were used only for information on whether a person was deceased. In some cases, no records were found. For 17 participants, residing outside the southern region, information of cause of death was not possible to obtain.

Motor symptoms were assessed with the Unified PD Rating Scale, UPDRS part III (score range 0–108, higher = worse).^28^ NMS were assessed with the NMS Scale (NMSS, score range 0–360, higher = worse).^29^ Cognition was assessed with the Mini-Mental State Examination (MMSE, score range 0–30, higher = better).^30^ Depressive symptoms were assessed with the Geriatric Depression Scale (GDS-30; score range 0–30, higher = worse).^31^

### Statistical analyses

Descriptive data are given as median with first and third quartiles (q1-q3) and frequencies and percentages, as appropriate. Associations were tested statistically with simple Cox regression analyses. For the multivariable analyses, eleven independent variables with *P*-values < 0.3 from the simple Cox regression analyses were simultaneously entered into a multivariable Cox regression model in order to identify factors independently associated with higher mortality hazard. A backward stepping regression analysis was conducted where *P*-values were inspected and the variable with the highest *P*-value was manually removed from the model, which was repeated until the remaining independent variables in the model had *P*-values < 0.1. P-values of < 0.05 were considered statistically significant. All analyses were performed using IBM SPSS version 30.0 (IBM 211 Corporation, Armonk, NY, USA). Kaplan Meier curves illustrating survival over time were generated using R version 4.5; R Core Team, 2025.

### Ethics statement

The study was approved by the Lund: Regional Ethics Review Board (Etikprövningsnämnden, EPN), Project number: JPND HC 559-002; Dnr: 2014-561 on Oct 28, 2014 and was performed in line with the principles of the Declaration of Helsinki. Written informed consent was obtained by the participants.

## Results

The cohort consisted of 107 patients, 62 (58%) of them male, median age 78 years, median disease duration 15 years and 79 (74%) of them were in HY stage IV at baseline assessment. Median age at disease onset was 63 years and at death 81 years. At baseline, 64 (60%) of the participants lived at home while the rest lived in a nursing home facility. Three years after baseline assessment, 52 (54%) of the participants were alive, after five years 30 (31%) and after seven years 14 (15%). At a final follow-up 7–9 years after the initial assessment, eight (8%) of the participants were alive (Table 1). Among the deceased at the final follow-up, 51 (57%) were male.

**Table 1.** Demographic and clinical data in relation to baseline assessment.

	Total N = 107	HY IV n = 79 (74%)	HY V n = 28 (26%)	Mis.
Gender, n (%)				
Male	62 (58%)	46 (58%)	16 (57%)	
Female	45 (42%)	33 (42%)	12 (43%)	
Age (yrs), median (q1-q3, min-max)	78 (73–84, 58–92)	78 (73–84, 63–91)	80 (76–84, 58–92)	
Age at onset (yrs), median (q1-q3, min-max)	63 (55–71, 30–82)	63 (55–72, 30–82)	64 (55–71, 50–78)	
Age at death (yrs), median (q1-q3, min-max)	81 (78–87, 67–96)	81 (78–87, 67–96)	82 (79–85, 69–90)	19
PD duration (yrs), median (q1-q3, min-max)	15 (11–19, 7–42)	14 (11–18, 7–42)	17 (11–20, 7–34)	
PD duration at death (yrs), median (q1-q3, min-max)	17 (14–22, 7–44)	17 (14–22, 7–44)	19 (13–22, 8–34)	19
Partner (yes), n (%)	65 (60%)	48 (61%)	17 (61%)	
ADL independency (%) (S&E), median (q1-q3, min-max)	40 (30–50, 10–80)	40 (40–50, 20–80)	20 (20–30, 10–40)	
Dwelling place, n (%)				
Home	64 (60%)	54 (68%)	10 (36%)	
Nursing home	43 (40%)	25 (32%)	18 (64%)	
Motor function (UPDRS III), median (q1-q3, min-max)	40 (29–53, 10–80)	34 (27–42, 10–73)	60 (50–70, 33–80)	
Non-motor symptomatology (NMSS), median (q1-q3, min-max)	91 (55–128, 20–223)	87 (51–111, 20–206)	125 (75–147, 41–223)	
Cognition (MMSE total score), median (q1-q3, min-max)	22 (18–27, 3–30)	23 (20–28, 3–30)	20 (13–25, 9–28)	4
Proportion ≥ 24, n (%)	43 (42%)			
≤ 23, n (%)	60 (58%)			
≤ 18, n (%)	27 (26%)			
Depressive symptoms (GDS-30) median (q1-q3, min-max)	11 (8–16, 2–30)	10 (8–16, 2–30)	13 (10–17, 2–27)	7
LEDD, median (q1-q3, min-max)	798 (560–998, 0–2300)	798 (560–965, 0–1826)	759 (556–1054, 400–2300)	
*Mortality data*				
Patients alive 3 yrs after baseline assessment	52/96 (54%)	44/70 (63%)	8/26 (31%)	11
Patients alive 5 yrs after baseline assessment	30/96 (31%)	28/70 (40%)	2/26 (8%)	11
Patients alive 7 yrs after baseline assessment	14/96 (15%)	14/70 (20%)	0	11
Patients alive at follow-up (7–9 yrs after baseline assessment)	8/97 (8%)	8/71 (11%)	0	10

Yrs: years; PD: Parkinson's disease; q1-q3: first and third quartiles; HY: Hoehn and Yahr staging scale (score range I-V, higher = worse); Mis.: missing; ADL: activities of daily living; S&E: Schwab & England ADL scale (score range 0–100, higher = better); UPDRS III: Unified PD Rating Scale, part III = motor examination (score range 0–108, higher = worse); NMSE: Non-Motor Symptoms Scale (0–360, higher = worse); MMSE: Mini-Mental State Examination (score range 0–30, higher = better); GDS-30: Geriatric Depression Scale (score range 0–30, higher = worse); LEDD: Levodopa (L-dopa) equivalent daily dose.

For patients who died in a hospital, the most common main cause of death recorded in the medical records was aspiration pneumonia (34%), followed by sepsis (16%), unspecified pneumonia (13%), ileus (5%) and Covid-19 (5%). Other main causes of death were: iliopsoas abscess, zoster encephalitis, intracerebral infarction, intracerebral hemorrhage, aortic dissection, cardiac arrest, pulmonary embolism with acute cor pulmonale and influenza (seasonal) (Table 2).

**Table 2.** Main causes of death in late stage PD when listed in the medical records (n* *=* *38).

	n (%)
Pneumonia and respiratory total, n = 21 of 38 (55%)	
Pneumonia total, n = 18 of 38 (47%)	
Aspiration pneumonia	13 (34%)
Pneumonia, unspecified	5 (13%)
Influenza (seasonal)	1 (2.5%)
Covid-19	2 (5%)
Sepsis total, n = 6 of 38 (16%)	
Sepsis unspecified	2 (5%)
Sepsis unspecified, colon ileus	1 (2.5%)
Sepsis unspecified, gastroenteritis and colitis, unspecified	1 (2.5%)
Sepsis, bowel perforation at laparoscopic surgery	1 (2.5%)
Urosepsis	1 (2.5%)
Ileus	2 (5%)
Bradycardia unspecific (possibly ileus or subileus)	1 (2.5%)
Iliopsoas abscess (due to stoma placement surgery)	1 (2.5%)
Zoster encephalitis	1 (2.5%)
Intracerebral infarction	1 (2.5%)
Intracerebral hemorrhage	1 (2.5%)
Intracerebral hemorrhage, post PEG removal surgery	1 (2.5%)
Aortic dissection	1 (2.5%)
Cardiac arrest	1 (2.5%)
Pulmonary embolism with acute cor pulmonale	1 (2.5%)

*PD: Parkinson's disease;* Covid-19: coronavirus disease 2019; *PEG:* Percutaneous Endoscopic Gastrostomy. When listed in the medical records, i.e., patients who were admitted to the hospital at time of death, n = 38. Out of the original cohort with 107 participants, 89 had died at the final follow-up, while eight were alive and 10 missing. For 17 participants, residing outside the health care region, information on main causes of death was not possible to obtain, due to not having access to their medical records.

**Figure 1.**
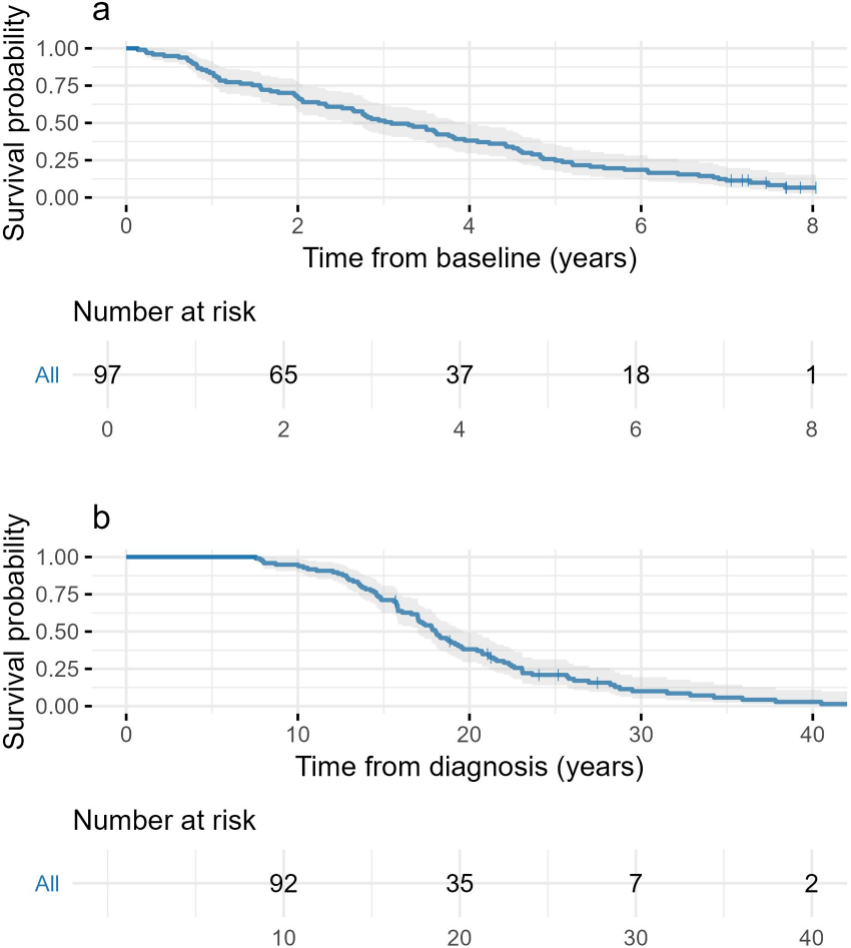
Kaplan Meier curves showing (a) survival from baseline assessment and (b) survival from PD diagnosis in a late stage PD cohort (n = 97).

In simple Cox regression analyses, baseline variables significantly associated with higher mortality hazard were identified as the following: worse ADL independency (*P* < 0.001), worse Hoehn and Yahr stage (*P* < 0.001), worse motor function (UPDRS III total score) (*P* < 0.001), worse cognition (MMSE total score) (*P* < 0.001), higher age (*P* = 0.005), worse perceptual problems/hallucinations (*P* = 0.020) and dwelling place (having moved to a nursing home) (*P* = 0.021) (Table 3). In a multivariable Cox regression model, worse motor function (*P* = 0.001), worse depressive symptoms (*P* = 0.023) and worse cognition (*P* = 0.042) were significantly associated with higher mortality hazard. Worse perceptual problems/hallucinations (*P* = 0.072) came close to being statistically significant in the multivariable model (Table 4). Kaplan Meier curves illustrate survival over time in this late stage cohort, both from baseline assessment and from PD diagnosis (Figure 1).

**Table 3.** Simple cox regression analyses (n = 97).

Independent variables	HR (95%CI)	*P*-value
Gender (male)	1.27 (.83, 1.95)	.274
Age (years)	1.05 (1.01, 1.08)	**.005**
PD duration (years)	1.01 (.98, 1.05)	.382
Partner (yes)	.98 (.64, 1.51)	.924
ADL independency (S&E)	.96 (.95, .98)	**<0.001**
Dwelling place (home vs nursing home)	1.65 (1.08, 2.53)	**.021**
HY stage	2.57 (1.59, 4.16)	**<0.001**
Motor function (UPDRS III)	1.04 (1.02, 1.05)	**<0.001**
Non-motor symptomatology (NMSS)	1.00 (.998, 1.01)	.390
*NMS domains*		
Cardiovascular including falls	1.00 (.97, 1.04)	.952
Sleep/fatigue	.996 (.97, 1.02)	.683
Mood/apathy	1.00 (.99, 1.02)	.612
Perceptual problems/hallucinations	1.04 (1.01, 1.07)	**.020**
Attention/memory	1.02 (.997, 1.03)	.094
Gastrointestinal tract	1.00 (.98, 1.03)	.798
Urinary	1.003 (.99, 1.02)	.721
Sexual function	1.01 (.99, 1.04)	.341
Miscellaneous	.99 (.97, 1.01)	.290
Cognition (MMSE)	.94 (.90, .97)	**<0.001**
Depressive symptoms (GDS-30)	.96 (.90, 1.02)	.190
LEDD (mg)	1.00 (.999, 1.00)	.873

HR: Hazard ratio; HY: Hoehn and Yahr staging scale (score range I-V, higher = worse); S&E: Schwab & England ADL scale (score range 0–100, higher = better); UPDRS III: Unified PD Rating Scale, part III = motor examination (score range 0–108, higher = worse); NMS: Non-motor symptoms; NMSS: Non-motor symptoms scale (0–360, higher = worse), NMS domains (severity×frequency of each item of the domain are added together: 0–12, higher = worse); MMSE: Mini-Mental State Examination (score range 0–30, higher = better); GDS-30: Geriatric depression scale (score range 0–30, higher = worse). Bold *P*-values statistically significant at *P* < 0.05.

**Table 4.** Multivariable cox regression analyses (n = 88).

Independent variables^1^	HR (95% CI)	*P*-value
Motor function (UPDRS III)	1.03 (1.01, 1.05)	**0.001**
Depressive symptoms (GDS-30)	.92 (.86, .99)	**0.023**
Cognition (MMSE)	.95 (.90, .998)	**0.042**
Perceptual problems/hallucinations (NMSS D4)	1.04 (.997, 1.08)	0.072

HR: Hazard ratio; UPDRS III: Unified PD Rating Scale, part III = motor examination (score range 0–108, higher = worse); NMSS: Non-motor symptoms scale (higher = worse), D: domain. ^1^ Independent variables entered in the multivariable Cox regression model (backwards method): gender; age; ADL independency (Schwab & England Scale); dwelling; Hoehn and Yahr stage; motor function (UPDRS III); NMSS domain 4 Perceptual problems/hallucinations; NMSS domain 5 Attention/memory; NMSS domain 9 Miscellaneous; cognition (MMSE); depressive symptoms (GDS-30). Bold *P*-values statistically significant at *P* < 0.05.

## Discussion

Having followed a large Swedish cohort of patients in late stage PD during the past decade has provided prospective, real-world data on mortality rate, main causes of death and factors associated with higher mortality hazard in late stage PD.

Out of the original cohort of 107 participants, just above half were still alive after three years, about a third after five years and only eight were still alive at a final follow-up 7–9 years after baseline assessment. Aspiration pneumonia was the most common main cause of death (34%) and when grouped together with unspecified pneumonia accounted for 47% of deaths in this cohort. Worse motor function, worse depressive symptoms and worse cognition were in a multivariable model associated with higher mortality hazard.

It has been suggested that late stage PD constitutes on average the last 4–5 years of the disease.^19,32^ In post-mortem studies, mean disease duration until death ranged from 6.9 to 14.3 years.^21^ In this fairly large cohort of patients in late stage PD, median (q1-q3) disease duration at baseline assessment was 15 (11–19) years. The majority (74%) of the participants were in HY stage IV at inclusion. PD patients in developed countries today might live slightly longer with the disease than two decades ago and quite a bit longer than they did 60 years ago before the initiation of levodopa therapy.^25^

It has been suggested that the prevalence of movement disorders is expected to increase in the coming decades due to aging populations and longer life expectancy.^33^ Factors such as availability of PD specialist care, infrastructure and a general prolonged life expectancy due to better living conditions are likely to continue to increase, though may vary between countries and regions of the world.

Long-term follow-up of patients with PD has previously been proposed in order to optimally investigate factors influencing mortality, as knowledge on predictors of poor prognosis may enable individually tailored interventions.^21^ In the present cohort, factors associated with higher mortality hazard were worse ADL independency, worse Hoehn and Yahr stage, worse motor function, worse cognition, higher age, worse perceptual problems/hallucinations and having moved to a nursing home. Worse perceptual problems/hallucinations came close to being statistically significantly associated with higher mortality hazard also in the multivariable Cox regression model. In a recent Spanish study, factors associated with mortality in hospitalized PD patients were higher age, comorbidities and male sex.^34^ Overall, increasing age and dementia have in a systematic review and meta-analysis been identified as the strongest factors associated with increased mortality in PD,^21^ where dementia in particular has been identified as a factor associated with increased mortality in PD.^23^ Both worse cognition and increasing age were among the factors most strongly associated with higher mortality hazard in PD also in the present cohort. The factors identified in the present cohort as associated with higher mortality hazard indicate that the worsened motor state and poorer everyday functioning of the late stage PD patients highly influence mortality hazard. All these factors, i.e., worse independency in ADL, higher Hoehn and Yahr stage, worse motor function, worse cognition, increasing age and perceptual problems/hallucinations contribute to the frail state of the late stage PD patient. In addition, difficulty swallowing, highly prevalent in late stage PD,^13,35–37^ contributes to an increased risk of aspiration pneumonia, which was the main cause of death in the present cohort.

Mortality in PD has previously been reported as higher than in non-PD controls.^21,23^ A British study including a large number of individuals with PD (10,000) and non-PD controls (55,500) showed that PD was associated with slightly increased mortality, which gradually increased with advancing disease.^38^ It has previously been shown that mortality increases with disease duration in PD^21^ and that reasons may be disease severity, complications of PD and comorbidities and therefore it has been suggested that complications and comorbidities need to be identified and addressed in order to reduce mortality.^38^ The results of the present analyses indicate that attention should be paid to the most common causes of death, in order to better identify risk areas for poor prognosis and optimize treatment and care for the late stage PD patient. The results showed that the most common main causes of death were aspiration pneumonia (34%), followed by sepsis (16%) and unspecified pneumonia (13%). It is likely to believe that among the cases of unspecified pneumonia, aspiration may have been the underlying cause, making the number of aspiration pneumonia even higher. If adding patients whose main cause of death was seasonal influenza and Covid-19, the number of deaths caused by respiratory problems was as high as 55%.

In the general population, the most common causes of death presently in Sweden have been listed as cardiovascular disease, followed by tumors.^39^ It is noteworthy that only five of the late stage PD patients of this cohort died from cardiovascular disease (intracerebral infarction, intracerebral hemorrhage [two cases; one as a complication of percutaneous endoscopic gastrostomy, PEG, removal surgery], aortic dissection, pulmonary embolism, cardiac arrest).

It has previously been shown that difficulty swallowing is a common symptom in late stage PD, affecting at least 40% of patients and being pronounced in around 25% of them (i.e., ≥ 6 points on NMSS item difficulty swallowing; each item 0–12 points, higher = worse).^13^ Swallowing difficulties are seen also in earlier disease stages,^36^ present in 39% of a cohort of 165 PD patients in stages I-IV, though pronounced only in 13% of the cases.^14^ This strongly supports the need for clinicians to be aware of the common occurrence of swallowing problems and screen for and treat swallowing problems.

It has been proposed that dysphagia is underrecognized and contributes to aspiration pneumonia, the leading cause of death in PD.^40,41^ Aspiration pneumonia is suggested to account for 25% of deaths among PD patients in the US, making it the most common cause of death.^41^ Based on the current results, there may be reason to believe that the number could be even higher.

Regular screening in the clinic is necessary to detect dysphagia and a multidisciplinary team approach has been suggested in order to identify and manage dysphagia.^40,41^ This could be done, e.g., through referral to a speech and language therapist for advice and instructions to the patients, their informal family caregivers and to personnel in nursing homes regarding suitable dietary adjustments (e.g., modified food texture, eating posture as well as a calm eating environment), in order to reduce the risk of aspiration.^40,41^ Although dementia has been shown to affect the ability to follow strategies to avoid aspiration,^42^ precautions such as an upright position when eating/drinking, the consistency of beverages (e.g., carbonation or thickening) and other food items could be taken by caregivers. Clinical guidelines suggest that people with PD with problems swallowing or managing saliva should have access to speech and language therapy interventions.^43,44^ Evidence-based interventions in this area may be somewhat limited. However, there have been attempts of methods including instruments designed to provide percutaneous electrical neck stimulation in order to enhance neuromuscular functioning improving the cough reflex. This has been suggested to be further evaluated in randomized controlled trials.^45^ Moreover, respiratory training interventions such as expiratory muscle strength training (EMST) have shown positive effects in significantly improving swallowing safety. Further standardization of the device, outcome measures and intervention protocols have been suggested to increase the level of evidence for the method.^46^

It has been suggested that the use of antipsychotic medications may increase the risk of aspiration pneumonia in older PD patients, due to dry mouth and impaired swallowing caused by drugs with anticholinergic effects.^47^ However, in the treatment of hallucinations and delusions, clozapine has been given high priority and recommended as clinically useful in national and international guidelines.^13,14,43,48^ This may be part of the reason why perceptual problems/hallucinations were associated with increased mortality in the present cohort. However, reasons for the limited use of clozapine^13,14^ are probably due to the risk of agranulocytosis, extensive monitoring requirements and regulatory and legal obstacles.^49^

Already in 2003, another Swedish study investigating causes of death in patients with PD followed for 9 years, reported respiratory causes (i.e., pneumonia in 28 of 29 cases) as the second largest cause of death in PD (24% among PD patients compared to 8% among controls without PD), after “other causes”. However, it was concluded that pneumonia was the most common cause of death in PD.^50^

Previous studies have shown that treatment is often not optimized and could be improved in order to reach better therapeutic outcomes also in the late stage of the disease.^2,11,12^ The overall situation for the late stage PD patient could likely be improved through optimizing treatment as well as through anticipating and better preventing situations at risk for this vulnerable group of patients, e.g., through better assessing, identifying and treating swallowing difficulties.

### Strengths, weaknesses and future perspectives

According to the literature, this is the first study where a large cohort of patients in late stage PD has been followed for almost a decade. This has provided valuable, observational, real-world clinical data and on mortality, main causes of death and factors associated with higher mortality hazard.

A major strength is that in addition to information on mortality rate and main causes of death, due to the extensive data material collected in this cohort, it was possible to carry out both simple and multivariable Cox regression analyses, including demographical variables as well as data on motor and non-motor symptomatology.

The majority of the participants, i.e., 89 (92%) were deceased at the final follow-up (> 7 years after baseline assessment). This number could presumably have been even higher, as there was no information to obtain on 10 individuals.

For 17 participants, residing outside the health care region, information on main causes of death was not possible to obtain, due to not having access to their medical records. Main causes of death were calculated among participants where these data were available.

Future studies in different international contexts and across various health care systems may provide additional information and insights. One should be aware of that there may be differences globally due to different degrees of socio-economic development when it comes to reporting of mortality rates and main causes of death as well as basic availability of health care resources and neurologists trained in movement disorders. Internationally standardized systems for reporting would be desirable, for future better globally representative and comparable data.^51^ Future perspectives for optimizing treatment and care for late stage PD patients may include a system with individual assessments and care coordination carried out by specially trained neuro-palliative PD nurses.^15,52^

## Conclusion

The present real-world, end-of-life data on late stage PD may provide treating clinicians with awareness and enhanced knowledge on factors associated with higher mortality hazard in this population, in order to better recognize these in the clinic and to optimize treatment and care. As aspiration pneumonia is the most common cause of death in PD worldwide and swallowing difficulties with the risk of aspiration are common in late stage PD, enhanced focus on prevention and treatment is essential and may improve the situation for these patients. The results emphasize the importance of clinicians being aware of, screening for and optimizing treatment for swallowing problems in late stage PD. Knowledge on mortality rates, main causes of death and factors associated with higher mortality hazard may contribute to better understanding of and care for late stage PD.
